# Supplementary material for: The Effect of Sexual Intercourse during Pregnancy on Preterm Birth: Prospective Single-Center Cohort Study in Japan
Source: Healthcare (Basel). 2023 Jun 5;11(11):1657. doi: 10.3390/healthcare11111657 (PMC10252402; doi:10.3390/healthcare11111657)
Supplement: Supplementary file 1 [file healthcare-11-01657-s001.zip › healthcare-2331899-supplementary.pdf]

Supplementary figure S1

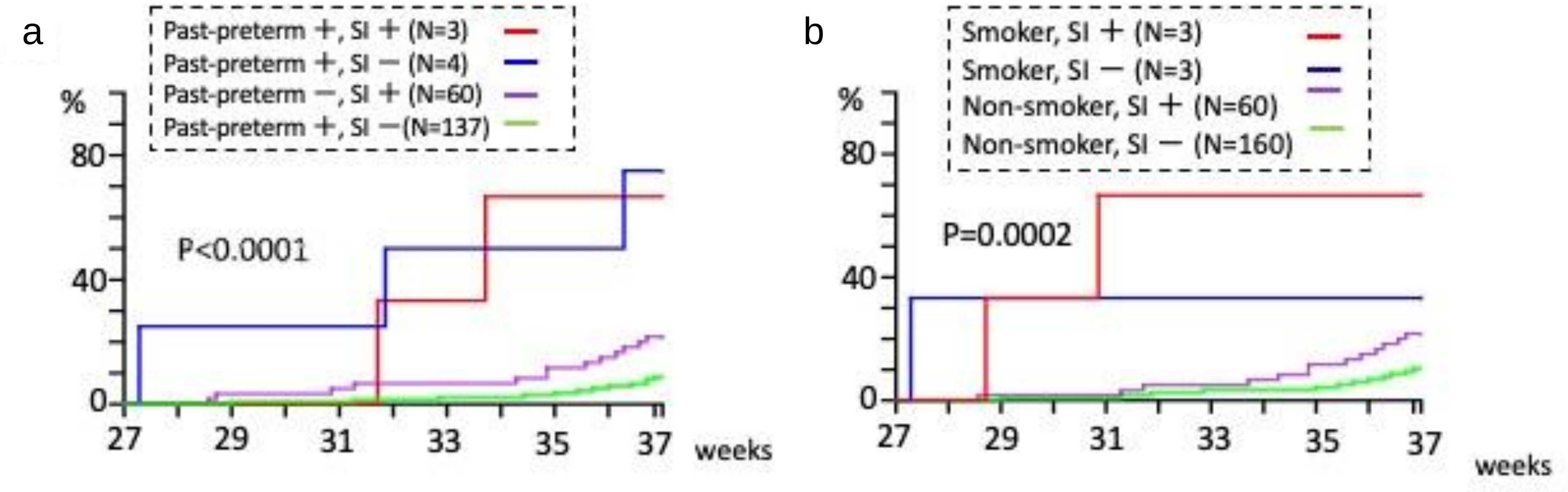

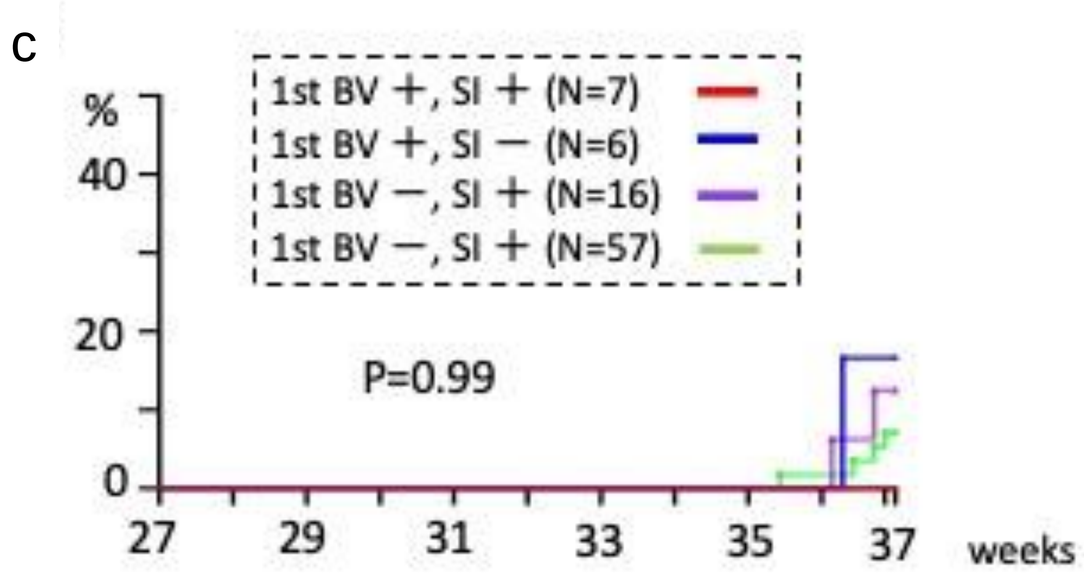

Supplementary Figure 1
Combination of SI with other risk factors and its association with cumulative preterm birth rate.
A) Combination with past history of preterm birth.
B) Combination with smoking during pregnancy.
C) Combination with BV in the first trimester.
The vertical and horizontal axes show the cumulative preterm birth rate (%) and the gestational age at preterm birth (weeks). There were no synergistic effects as shown in Figure 3.

Supplementary figure S2

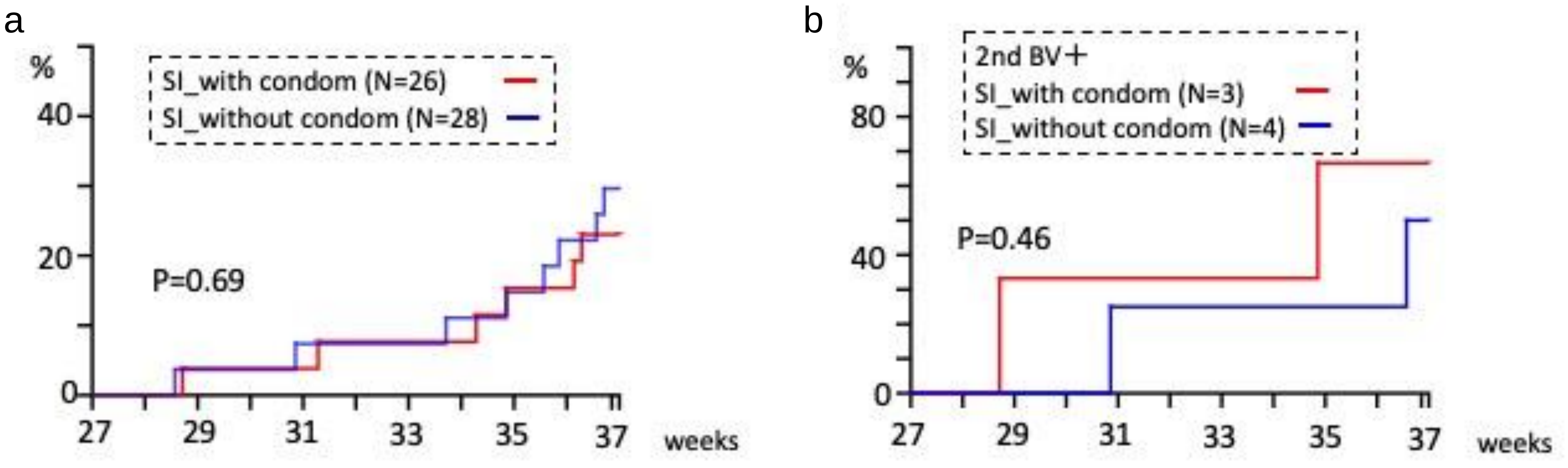

Supplementary Figure 2

The association between condom use and cumulative preterm birth rate.

A) Comparison of cases with and without condom use.

B) Comparison of BV cases in the second trimester with and without condom use.

Only cases with SI during pregnancy were analyzed.
